# Influence of Fatigue on Some Kinematic Parameters of Basketball Passing

**DOI:** 10.3390/ijerph18020700

**Published:** 2021-01-15

**Authors:** Feng Li, Damir Knjaz, Tomislav Rupčić

**Affiliations:** Laboratory for Sports Games, Faculty of Kinesiology, University of Zagreb, 10000 Zagreb, Croatia; damir.knjaz@kif.unizg.hr (D.K.); tomislav.rupcic@kif.unizg.hr (T.R.)

**Keywords:** angular velocity, accuracy, pelvis, ball speed

## Abstract

Kinematic analysis is an objective method for examining basketball technique. However, there are just a few studies featuring a kinematic analysis of passing. The purpose of this study was to determine whether the kinematic parameters and accuracy of passing changed when players were under the influence of fatigue. Eleven Croatian basketball players who are members of the youth national program (age: 18.36 ± 0.67 years; height: 192.32 ± 9.98 cm; weight: 83.35 ± 11.19 kg; body fat: 15.00 ± 4.40%, arm span: 194.34 ± 10.39 cm) participated in fatigue and non-fatigue repetitive tests. A Xsens suit was used to analyze the kinematic parameters of push passing; a radar gun was used to determine ball speed; heart rate and blood lactate were used to identify fatigue and non-fatigue state. There was a significant difference in angular velocities of shoulder (*p* = 0.01), elbow (*p* = 0.04), and wrist (*p* = 0.01), accuracy (*p* = 0.01), ball speed (*p* = 0.00), pelvis position (*p* = 0.00), and velocity of the pelvis in X-axis (*p* = 0.00) between fatigue and non-fatigue state. Fatigue influences some kinematic parameters and accuracy of passing. The findings of this study suggest that coaches conduct as many drills as possible in situational conditions that are similar to the conditions during the basketball game itself.

## 1. Introduction

The fundamental skills are the foundation for success at all levels of basketball, and all players must learn to execute them properly and quickly in order to be successful [1,2]. In basketball, passing is one of the most frequently used techniques during a competitive game [3,4]. There was a study reporting that passing technique is even the optimal factor to determine all-star and non-all-star NBA players [5]. During the offense, players are required to keep possession of the ball and cooperate to create optimal shooting options. Teams that assist more are more likely to win the game [6]. On the other hand, reducing the number of turnovers (i.e., lost possession of the ball) increases the chances of winning, especially in games where opponents have similar chances of winning [7]. Another aspect stated that players can have at least 50% efficiency in shooting but must have 100% in passing the ball [8].

Therefore, given the importance of passing in basketball skills, coaches should be persistent in developing passing skill.

From a physiological point of view, basketball is an intermittent sport that involves both intensive brief movements (e.g., jumping, sliding, and sprinting) and less intensive long-lasting activities (e.g., walking and running). Thus, the players’ physiological demands of a basketball game, which require both aerobic and anaerobic energy systems, are claimed to be high [9]. Some studies found that the blood lactate levels, mean heart rates, and VO_2_ max of the players during a competitive game were close to their maximal values [10,11,12]. 

Fatigue becomes an unavoidable part of the game that may deteriorate performance, coordination, and the players’ technique [13]. Some players have highly developed skills, but their quality of performance can be impaired under the influence of fatigue, which will consequently impair their efficiency in the game. The changes of kinematic parameters under the influence of fatigue in basketball (e.g., trajectory of ball flight, joint angles in upper and lower extremity, and center of mass) have generally been investigated when shooting, where authors proved that fatigue can affect changes in some kinematic parameters when shooting from different playing positions [14,15,16]. 

On the other hand, there are only a few studies that investigated the change of passing accuracy under fatigue [17,18]. However, these studies mainly focused on passing accuracy or ability, while no kinematic parameters were observed. Thereby, certain movement patterns of passing in different conditions still remain mostly unexplored. 

The kinematic analysis represents an objective method to observe basketball players’ passing skills [19], which can provide a scientific explanation for mistakes in passing performance, especially under fatigue. In a study by Theoharopoulos et al [20], chest pass, overhead, and push pass were the most commonly used in basketball games, with the latter having importance when the players face defense pressure. However, there is no research focusing on the push passing in terms of passing accuracy and kinematic analysis under fatigue. Therefore, the aim of this study is twofold: (i) to examine whether the kinematic parameters of push passing changed when a player is under the influence of fatigue; (ii) to identify if the fatigue affects the passing accuracy. We hypothesized that the kinematic parameters would be changed, and the passing accuracy would be decreased when a player is under the influence of fatigue.

## 2. Materials and Methods 

### 2.1. Participants 

The sample consisted of 11 Croatian basketball players (age: 18.36 ± 0.67 years; height: 192.32 ± 9.98 cm; weight: 83.35 ± 11.19 kg; body fat: 15.00 ± 4.40 %, arm span: 194.34 ± 10.39 cm) who are members of the Croatian youth national program. Players had no health nor injury issues. In order to avoid the interference of fatigue on testing, players were asked to restrain from training sessions one day before testing. All participants were provided with a detailed explanation of the study procedures and gave written informed consent prior to the measuring procedure. The Faculty of Kinesiology, University of Zagreb (Croatia) Ethics Committee approved the study, which was performed following the ethical standards of the Declaration of Helsinki. 

### 2.2. Experimental Procedures

Each player was tested in one day, but the overall testing was conducted during three days in the following manner: three players were tested on the first day, and the other eight participants were tested on the following two days (i.e., four players on each day). All players underwent the same protocol: before testing, they had one day of rest, while the testing performed the next day consisted of warm up, non-fatigue passing testing, fatigue protocol, and fatigue passing testing. Basic anthropometric characteristics were measured on each day of the testing and used for system calibration performed according to the instruction of the manufacturer (Xsens technologies B.V., Netherlands). In order to identify the level of players’ fatigue, their Heart Rate (HR) and Blood Lactate (BL) were measured by heart rate sensors (Polar H10, manufacturer: Polar, Kempele, Finland) and a portable lactate analyzer (Lactate Scout 3, manufacturer: SensLab GmbH, Leipzig, Germany) respectively before starting the test. In addition, the participants’ HR and BL were measured once more immediately after conducting the fatigue protocol. Then, the same passing test was conducted again in order to observe the change of passing accuracy and kinematic parameters under the influence of fatigue. 

The following variables were observed: pelvis position from the point the player caught the ball until release (PELVIS_P) (cm); pelvis velocity in X-axis from the point the player caught the ball until release (PELVIS_X_axis_) (m/s); pelvis velocity in Y-axis from the point the player caught the ball until release (PELVIS_Y_axis_) (m/s); maximum angular velocities in shoulder from the point the player started to pass the ball until release (SHOULDER_AV_max_) (°/s), maximum angular velocities in elbow from the point the player started to pass the ball until release (ELBOW_AV_max_) (°/s), and maximum angular velocities in wrist from the point the player started to pass the ball until release (WRIST_AV_max_) (°/s); the speed of ball approaching the target (BALL_S) (km/h); and the passing accuracy (ACCURACY) (points).

To measure kinematic variables, the Xsens MVN inertial suit system was used with 17 three dimensional accelerometers/gyroscopes/magnetometers. The kinematic parameters of push passing were derived from the corresponding MVN BIOMECH software (MVN Studio 4.4, firmware version 4.3.1). Previous study has confirmed the reliability and validity of Xsens kinematic suit for analyzing angular velocity and other kinematic parameters in different basketball techniques [21]. In addition, it was used in previous study for measuring similar data in the field of basketball [22]. The ball speed was measured by a radar gun (Stalker ATS 2, manufacturer: Stalker Sport, Texas, USA) with its reliability being previously proved in the sport field [23,24]. 

### 2.3. Test Protocol

**Figure 1:**
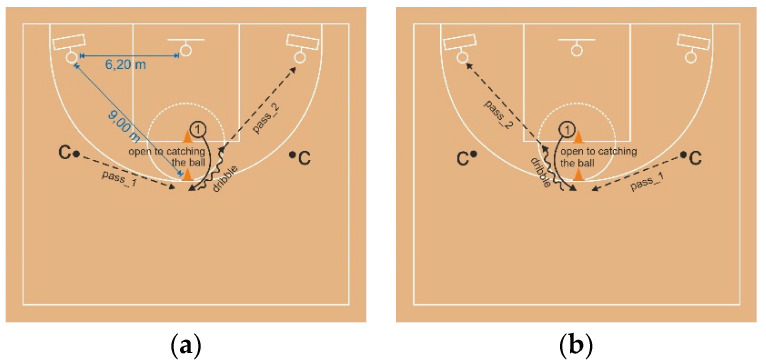
(**a**) push pass on the right side; (**b**) push pass on the left side.

**Figure 2:**
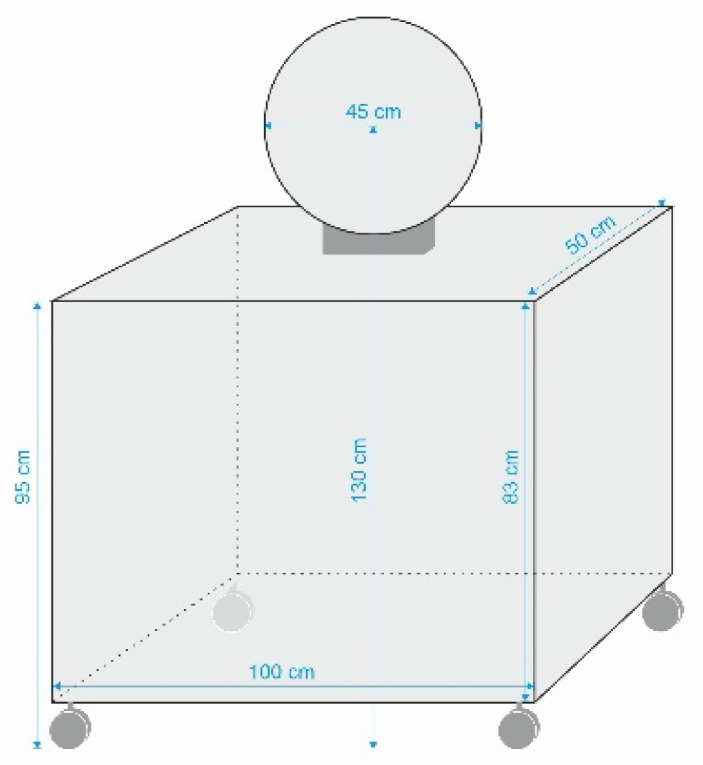
Sketch of the target.

As shown in Figure 1 and Figure 2, two hoops were placed vertically near the left and the right court corner 1.30 m above the ground. The distance between the hoops and the basket was 6.20 m, and the distance between the hoops and the top of the three-point line was 9 m. Participants were standing in the middle of the free throw line with their back to the basket. Two basketball players were standing on the wing of both the left and the right side, passing the ball to the participant. The participant ran to the top of the three-point line and received the ball; then, they did a crossover with one dribble and passed the ball with the right hand toward the right target (hoop). After passing to the right target, the player ran to the left side and repeated the same task. Players executed six passes to the right and left sides, respectively. Due to some technical issues with equipment and motor movement, there were several passes that were not taken for further analysis. Prior to the test, the players had three trial passes. The warmup consisted of 5 min of jogging and 5 min of specific stretching. According to a previous study [18], the test scoring was as follows:

Eight points were awarded for each pass that hit the target without touching the hoop.

Six points were awarded for each pass that hit the target but touched the hoop once.

Four points were awarded for each pass that hit the target but touched the hoop more than once.

Two points were awarded for each pass that did not hit the target but touched the hoop.

No points were awarded if the ball did not hit the target nor touch the hoop, or if a pass other than a push pass was used.

### 2.4. Fatigue Protocol

The 300-meter shuttle run (15 × 20 m^2^ with the change of direction of 180°) was used as fatigue protocol due to similarities with game situations in which a player runs forward and backwards consecutively. The reliability of this fatigue protocol was previously verified [25,26]. Players were instructed to sprint as fast as possible during the fatigue protocol, and the running time was recorded by photocells (WittyGate, manufacturer: Microgate, Bolzano, Italy).

### 2.5. Data Analysis

“Statistica” version 13.5.0.17 (TIBCO Software Inc, Palo Alto, CA, USA; release date: November 2018) was used for the statistical analysis. Basic descriptive parameters were calculated for all measured variables. The normality of the data distribution was evaluated using Kolmogorov–Smirnov test. To verify the differences of the kinematic parameters between fatigue and non-fatigue, analysis of variance (ANOVA) for repeated measures was applied. With the use of the G*power program, the sample size (number of passes) was calculated (n = 98) that was needed for measurement procedure with statistical significance *p* ˂ 0.05; statistical power 0.8; effect size 0.25 and groups.

## 3. Results

**Figure 3:**
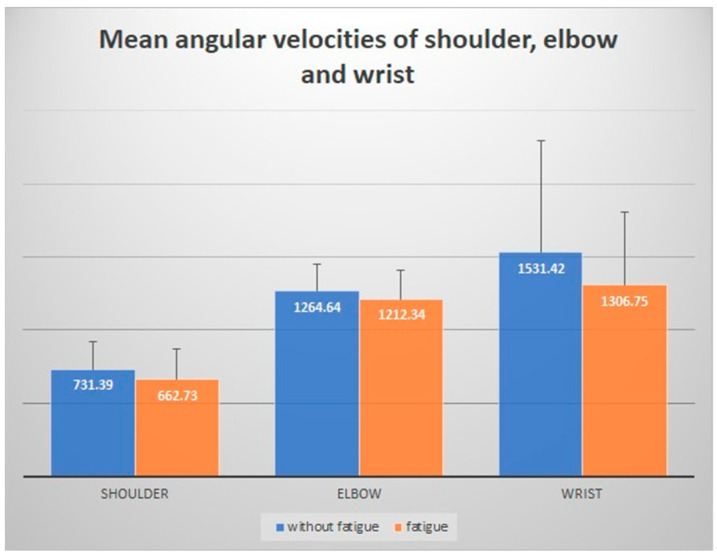
Comparison of angular velocities between fatigue and non-fatigue.

As shown in Table 1, the mean value of passing accuracy (ACCURACY) when players were under the influence of fatigue was decreased compared to non-fatigue condition (fatigue = 1.93; non-fatigue = 2.81); the mean value of maximum angular velocity in non-fatigue condition was higher than fatigue condition, regardless of shoulder (SHOULDER_AV_max_) (non-fatigue = 731.39; fatigue = 662.73), elbow (ELBOW_AV_max_) (non-fatigue = 1264.64; fatigue = 1212.35), and wrist joint (WRIST_AV_max_) (non-fatigue = 1531.42; fatigue = 1306.75). The mean value of maximum angular velocity in wrist joint (WRIST_AV_max_) was obviously higher than in elbow (ELBOW_AV_max_) and shoulder (SHOULDER_AV_max_) both in fatigue and non-fatigue conditions (Figure 3); the mean value of pelvis position (PELVIS_P) when players were under fatigue was increased compared to the non-fatigue condition (fatigue = 0.93; non-fatigue = 0.89); the pelvis velocity in X-axis (PELVIS_X_axis_) and Y-axis (PELVIS_Y_axis_) when players were under fatigue was lower than in non-fatigue condition; the ball speed (BALL_S) when players were under fatigue was decreased compared to non-fatigue condition (fatigue = 39.96; non-fatigue = 42.38).

In addition, the results in Table 1 indicated that there was a significant difference between fatigue and non-fatigue conditions in angular velocities in terms of shoulder (SHOULDER_AV_max_; *p* = 0.01), elbow (ELBOW_AV_max_; *p* = 0.04) and wrist (WRIST_AV_max_; *p* = 0.01). In addition, there were significant differences in passing accuracy (ACCURACY; *p* = 0.01) and ball speed (BALL_S; *p* = 0.00) between fatigue and non-fatigue conditions; there were significant differences in the pelvis position (PELVIS_P; *p*= 0.00) and the pelvis velocity X-axis (PELVIS_X_axis_; *p* = 0.00) between fatigue and non-fatigue conditions. However, there was no significant difference in the pelvis Y-axis (PELVIS_Y_axis_; *p* = 0.12).

As presented in Table 2, the mean values of players’ HR and BL under the influence of fatigue were obviously higher than in non-fatigue condition (fatigue: HR = 186.82; BL = 10.35; non-fatigue: HR = 87.82; BL = 1.45).

Table 3 shows that there was significant difference in push passing between the fatigue and non-fatigue conditions (F = 9.65, *p* = 0.00).

## 4. Discussion

There were few studies observing the change of kinematic parameters and accuracy of basketball passing under physiological load. The present study aimed to identify whether some kinematic parameters of push passing changed when players were under the influence of fatigue and to examine if fatigue affected the passing accuracy. The main findings from this study showed that there were significant differences between non-fatigue and fatigue conditions in kinematic parameters and passing accuracy, which is in line with our previously formulated hypotheses.

Physiological variables such as HR and BL can be measured not only in sterile laboratory conditions but also on field and in more authentic conditions [27]. HR and BL have been used in many studies to monitor the level of athletes’ fatigue [28,29,30,31]. In this study, the mean value of HR was 186.82 beats/min and the BL was 10.35 mmol/l during the testing. McInnes et al. [11] investigated the intensities of a real basketball competition by using a heart rate monitor. The results showed that the highest value of HR during the game was 188 beats/min. Another research by Abdelkrim et al. [10] reported that the highest BL concentration in a basketball game was 13.2 mmol/l varying from the player’s position and the level of competition. Consequently, it can be concluded that the level of players’ fatigue in this study was at the level of players’ fatigue in the actual situational conditions during the game. 

The results from this study showed that there were significant differences between non-fatigue and fatigue conditions in kinematic parameters. Slawinski et al. [16] investigated the influence of fatigue on the change of kinematics and accuracy in basketball shooting. They reported that the fatigue decreased hip joint angle and increased shoulder joint angle. Another two studies focused on the effect of progressive fatigue on the kinematic change of the longer shooting distance [14,15]. Their results showed that fatigue had a great influence on all kinematic parameters measured in their study. Therefore, the results of this study were similar to their results, which means that fatigue had a great influence on important kinematic parameters in performing basic elements of basketball technique. 

There was also a significant difference in passing accuracy and the speed of the ball approaching the target. A few studies investigated the change of passing accuracy related to fatigue. There were two studies investigating the impact of fatigue on the accuracy of basketball chest passing, and their results showed that there was a significant difference in passing accuracy when players were under the influence of fatigue [17,18]. The result of the present study is consistent with previous studies. 

In this study, the PELVIS_P in fatigue condition was significantly higher than in non-fatigue condition. Some previous studies have stated that decreases of lower limb muscle activation due to fatigue could result in changes in PELVIS_P, and the reduction of strength tended to increase the player’s center of mass [32,33]. Lafond et al. [34] pointed out that the higher center of mass may reduce the ability of balance, and Boccolini et al. [35] reported that the role of postural balance appeared to be important in shooting performance. Given that, it could be assumed that higher PELVIS_P implied a lower level of balance during passing performance, and the poor balance could consequently lead to a decrease of the passing accuracy. 

Fatigue can cause the reduction in the capacity of the muscle to generate force, which results in a player who is unable to continue moving at the same level of performance [15,36,37]. In addition, Kauranen et al. [38] reported that the increase in the strength of the upper extremities improved the coordination and velocity of movement. In this study, the value of upper extremity angular velocity, accuracy, and speed of the ball approaching the target in fatigue condition were greatly decreased compared to non-fatigue. This can be explained by the fact that fatigue affected the reduction of muscle strength and coordination, which ultimately reduced angular velocities in individual joint systems, the accuracy of the pass, and the speed of the ball during the pass.

**Figure 4:**
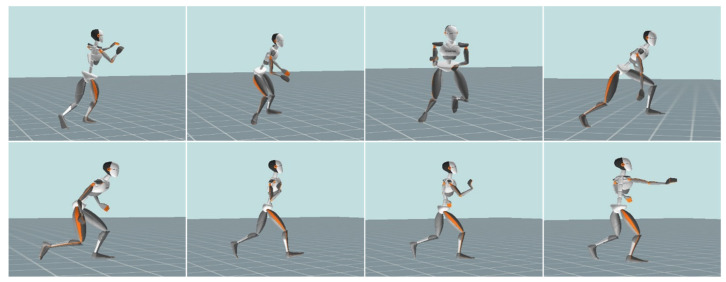
Kinogram from the point of catching the ball until releasing it from the wrist.

It was clear that the mean value of WRIST_AV_max_ was higher than ELBOW_AV_max_, and that of ELBOW_AV_max_ was higher than SHOULDER_AV_max_ both in fatigue and non-fatigue conditions (Figure 3). The body segments move in a certain sequence for multiple joint movements—the force is transmitted from the proximal to the distal body parts [39,40]. In addition, there were some studies that stated that greater velocities during shooting were associated with the strategies of the reuse of the energy transferred from the lower extremity to upper extremity [41,42,43,44]. Similarly, in the technique of push passing, the sequence of movement of the upper extremity is shoulder, elbow, and wrist (Figure 4), and the force of wrist joint is transferred from elbow and shoulder. The previously mentioned sequence of movement is constant regardless of players’ physiological condition. Components that can vary depending on influence of fatigue are values of forces and angular velocities. 

The PELVIS_X_axis_ was higher than in PELVIS_Y_axis_ both in fatigue and non-fatigue conditions, which reflected that the players were using correct technique during the testing. In order to pass by the defender, the drive needs to be performed in the direction of the basket [45]. Thus, the velocity in the direction of forward (X) should be higher than the direction of side (Y) that is mainly for creating space for drive toward the basket. The results in this study showed that there was a significant difference in PELVIS_X_axis_ (*p* = 0.00) between fatigue and non-fatigue conditions. It is very important to perform crossover with a quick first step after receiving the ball so that the offensive player can take an advantage over the defensive player. In that situation, another defensive player on the helping side tries to stop the player’s drive to the basket. That movement creates a situation for passing to an open offensive player who stays at the corner and waits for the pass and open shot or drive. In this study, the PELVIS_X_axis_ was decreased after fatigue protocol, which means that the player did not have enough velocity to pass by the defender to the basket and probably would not attract the help of the defender. As a result of that, the offensive player who stayed at the corner lost the opportunity to catch the ball and shoot.

### Limitations

The presented study focused on the technique of push passing, but different kinds of passing can be used in a game as well. Thus, it is worth exploring the change of kinematic parameters on other passing techniques such as chest or overhead passing. In addition, the limitation of this study was the situation that the testing was performed without defensive players who can be included in some future research. 

Therefore, these factors are worth analyzing in the future studies related to passing technique.

## 5. Conclusions

There were significant differences in maximum angular velocity of shoulder, elbow, and wrist between fatigue and non-fatigue. The passing accuracy and ball speed when players were under the influence of fatigue were significantly decreased compared to non-fatigue condition. The players’ pelvis position was obviously increased when they were under fatigue. There was a significant difference in pelvis velocity related to X-axis between fatigue and non-fatigue; however, there was no significant difference in Y-axis. The findings of this study could also help coaches better understand the pattern of movement of push passing and correct players’ technique.

It is extremely important that players adopt the correct motor structure of passing to create an automatism during the training process of learning, which will ultimately not change even under the influence of fatigue. Only this can ensure the situational efficiency of the player, because any deviation from the ideal biomechanical structure also affects the occurrence of a larger number of motor errors, and consequently reduced efficiency. 

From the aspect of cooperation between two players in offense, in addition to the correctly adopted movement structure, it is also necessary to perfect spatial–temporal relations in passing and catching the ball, which is possible if the conditions of the players’ training process are similar to the conditions of the game. 

## Figures and Tables

**Table 1:** Descriptive parameters and result of ANOVA for repeated measures of the fatigue and non-fatigue conditions.

Variable	Group	N	Mean	Min	Max	Std.Dev.	F	*p*
**SHOULDER_AV_max_ (°/s)**	Non-fatigue	114	731.39	398.63	1313.97	192.23	6.59	0.01 *
	Fatigue	114	662.73	261.04	1375.02	211.27		
**ELBOW_AV_max_ (°/s)**	Non-fatigue	114	1264.64	884.77	1811.54	183.12	4.31	0.04 *
	Fatigue	114	1212.35	454.63	1674.74	196.87		
**WRIST_AV_max_** **(°/s)**	Non-fatigue	114	1531.42	436.90	4476.40	766.39	6.91	0.01 *
	Fatigue	114	1306.75	390.11	3010.93	495.51		
**PELVIS_P (cm)**	Non-fatigue	114	0.89	0.73	1.00	0.068	18.02	0.00 *
	Fatigue	114	0.93	0.79	1.17	0.09		
**PELVIS_X_axis_** **(m/s)**	Non-fatigue	114	2.96	0.47	4.32	0.76	13.05	0.00 *
	Fatigue	114	2.54	0.10	4.10	0.96		
**PELVIS_Y_axis_ (m/s)**	Non-fatigue	114	2.12	0.20	3.87	0.72	2.50	0.12
	Fatigue	114	1.98	0.40	3.44	0.62		
**BALL_S (km/h)**	Non-fatigue	114	42.38	33.70	56.00	4.25	15.04	0.00 *
	Fatigue	114	39.96	24.00	58.00	5.15		
**ACCURACY** **(points)**	Non-fatigue	114	2.81	0.00	8.00	2.80	6.71	0.01 *
	Fatigue	114	1.93	0.00	8.00	2.29		

* Marked values were significant when *p* < 0.05. Legend: SHOULDER_AV_max_: maximum angular velocity of shoulder joint from the point the player started to pass the ball until release; ELBOW_AV_max_: maximum angular velocity of elbow joint from the point the player started to pass the ball until release; WRIST_AV_max_: maximum angular velocity of wrist joint from the point the player started to pass the ball until release; PELVIS_P: the position of player’s pelvis from the point the player caught the ball until release; PELVIS_X_axis_: the velocity of pelvis in X-axis from the point the player caught the ball until release; PELVIS_Y_axis_: the velocity of pelvis in Y-axis from the point the player caught the ball until release; BALL_S: the speed of the ball approaching to the target; ACCURACY: the passing accuracy.

**Table 2:** Descriptive statistics of Heart Rate (HR) and Blood Lactate (BL) when players were under different physiological load.

Variable	N	Mean	Minimum	Maximum	Std.Dev.
**HR_non_F (beats/min)**	11	87.82	68.00	106.00	11.05
**Max_HR_non_F (beats/min)**	11	179.55	165.00	200.00	11.12
**HR_F (beats/min)**	11	186.82	170.00	201.00	9.04
**Max_HR_F (beats/min)**	11	182.18	169.00	196.00	8.22
**BL__non_F (mmol/l)**	11	1.45	0.80	1.90	0.36
**BL_F (mmol/l)**	11	10.35	7.10	13.30	2.18
**300 m shuttle run (s)**	11	74.87	68.36	84.27	5.13

Legend: HR_non_F: The players’ heart rate in non-fatigue condition; Max_HR_non_F: The players’ maximum heart rate in non-fatigue condition during passing; HR_F: The players’ heart rate under the influence of fatigue; Max_HR_F: The players’ maximum heart rate under the influence of fatigue during passing; BL_non_F: The players’ blood lactate in non-fatigue condition; BL_F: The players’ blood lactate after the fatigue protocol.

**Table 3:** Result of ANOVA for repeated measures (for groups).

Test	Value	F	*p*
**Wilks**	0.74	9.65	0.00 *

* marked values were significant when *p* < 0.05.

## Data Availability

The data presented in this study are available on request from the corresponding author. The data are not publicly available due to its huge size and participants’ privacy protection.
